# Epidemiological review of leprosy in WHO’s Western Pacific Region: 1991–2019

**DOI:** 10.5365/wpsar.2021.12.3.858

**Published:** 2021-08-23

**Authors:** Kalpeshsinh Rahevar, Fukushi Morishita, Kyung Hyun Oh, Tauhid Islam

**Affiliations:** a World Health Organization Regional Office for the Western Pacific, Manila, Philippines.

## Abstract

**Background:**

Leprosy elimination was achieved in the Western Pacific Region of the World Health Organization (WHO) in the late 1980s. However, cases continue to be reported within the Region. This paper analyses leprosy cases in the Region reported to WHO during 1991–2019.

**Methods:**

Descriptive analyses were conducted of data from leprosy surveillance reported in the Region. Key indicators included prevalence, the number and rate of new cases detected, proportions of cases with multibacillary leprosy or grade 2 disability, and the numbers and proportions of cases among children and cases by sex.

**Results:**

From 1991 to 2019, the number of registered cases detected in the Region decreased by 94% (from 68 313 in 1991 to 4381 in 2019), and the number of new cases detected decreased by 72.1% (from 15 002 in 1991 to 4004 in 2019). The proportion of cases of multibacillary leprosy increased from 67.4% (8045/11 943) in 1995 to 85.6% (3428/4004) in 2019, and between 1997 and 2019 the number of leprosy cases occurring in children decreased from 1240 to 424. The proportion of new cases with grade 2 disability decreased from 15.4% in 1997 to 6.6% in 2019. With few exceptions, nearly two thirds of reported cases occurred in males.

**Conclusion:**

Most countries and areas in the Region have successfully eliminated leprosy, although some pockets remain in countries with hard-to-reach areas. The introduction of multidrug therapy and WHO’s 1991 elimination goals may have prompted the initial decline in leprosy cases. Continued efforts are required in case-finding, care and prevention in areas with a high burden of disease.

Leprosy, also known as Hansen’s disease, is a chronic infectious disease caused by *Mycobacterium leprae*. Depending upon the bacillary load, the disease is classified as paucibacillary (PB) or multibacillary (MB) leprosy. Early diagnosis and treatment prevent lifelong disabilities and deformities. (*1*)

In 1982, the World Health Organization (WHO) recommended the use of multidrug therapy (MDT) to treat both MB and PB leprosy. (*2*) In WHO’s Western Pacific Region, implementation of MDT began in 1985, and full coverage was achieved by 1994. (*3*) In 1991, the World Health Assembly endorsed resolution WHA 44.9 aimed at eliminating leprosy as a public health problem by the year 2000. (*4*) Elimination was defined as reducing the prevalence of registered leprosy cases to < 1/10 000 population.

Globally, in 2019 the number of registered leprosy cases was 177 175 and the number of new cases reported was 202 185. This translates to a global prevalence of 0.22/10 000 population and a new case detection rate (NCDR) of 2.59/100 000 population. Of the total number of new cases (202 185) reported in 2019, 68.9% (143 787) were reported by WHO’s South-East Asia Region, followed by 14.3% (29 936) from the Region of the Americas, 9.7% (20 205) from the African Region and 2% (4211) from the Eastern Mediterranean Region, with the Western Pacific Region accounting for 1.9% (4004) of new cases. (*5*)

The leprosy elimination target was achieved at the global level in 2000 (*6*) and at the national level in most countries by 2005. The Western Pacific Region, which consists of 37 countries and areas, achieved the leprosy elimination target at the regional level before 1988. However, a few small Pacific Island countries have not reached this target. During the two decades since 2000, four 5-year global leprosy strategies were implemented that aimed at improving the availability and accessibility of leprosy services and ending leprosy worldwide.

The objective of this study was to analyse the data on leprosy reported to WHO from 1991 to 2019 by countries and areas in the Western Pacific Region.

## Methods

### Definitions

A case of leprosy is a person who has yet to complete a full course of treatment and who has one or more of the following: (1) a definite loss of sensation in a pale (hypopigmented) or reddish skin patch; (2) a thickened or enlarged peripheral nerve, with a loss of sensation or with weakness of the muscles supplied by that nerve, or both; or (3) acid-fast bacilli on a slit-skin smear. (*7*) A PB case is defined as a person who has one to five skin lesions without demonstrated presence of acid-fast bacilli on a slit-skin smear. An MB case is defined as a person with leprosy who has more than five skin lesions or has nerve involvement (either pure neuritis or any number of skin lesions and neuritis) or who has the demonstrated presence of acid-fast bacilli on a slit-skin smear, irrespective of the number of skin lesions. (*1*)

The prevalence of leprosy was defined as the total number of leprosy cases registered for treatment in a given population at one point in time (usually the end of the reporting year) divided by the mid-year population and expressed as a rate per 10 000 population. (*6*)

The NCDR was defined as the number of new cases detected in a given population in a year, expressed as a rate per 100 000 population. (*6*)

Grade 2 disability (G2D) was defined as the presence of visible deformity or damage to the hands or feet, or severe visual impairment caused by leprosy. The rate of G2D was defined as the number of new cases with G2D detected among the new cases of leprosy (never treated before) in a defined population in a year, expressed as a rate per 1 million population. (*6*)

### Data sources

Leprosy surveillance data reported annually from each country and area of the Western Pacific Region to WHO were analysed. (*5*, *8*) These data include the number of registered cases and new cases by sex and disease type, the number of children aged < 15 years, and the number of cases with G2D. In 2019, 29 countries and areas reported, representing more than 99% of the total population of the Region. We requested that national leprosy programmes in 36 countries and areas in the Region (all except the Pitcairn Islands, which has an extremely small population) review and validate their historical data from 1991 to 2014, of which 14 (39%) complied. Population data used for calculating prevalence and NCDRs were sourced from the United Nations Population Division. (*8*)

### Analysis

Descriptive analyses of leprosy cases in the Region and by country and area were conducted, with key programme indicators calculated for the period between 1991 and 2019. These indicators included prevalence and NCDRs, proportions of cases with MB leprosy and G2D, proportions of cases occurring in children and females, and the rate of new cases with G2D.

Annual NCDRs in the Region were assessed for 1991–2019. The number and rate of new cases detected were compared for the countries with the highest number of leprosy cases. Owing to issues with completeness of data, the annual NCDRs stratified by disease type were assessed for 1995–2019, for children and G2D for 1997–2019, and for sex for 2007–2019.

### Ethics statement

Ethical clearance was not required because this report used routinely available data and no personal identifying information was collected.

## Results

### Prevalence

**Figure 1:**
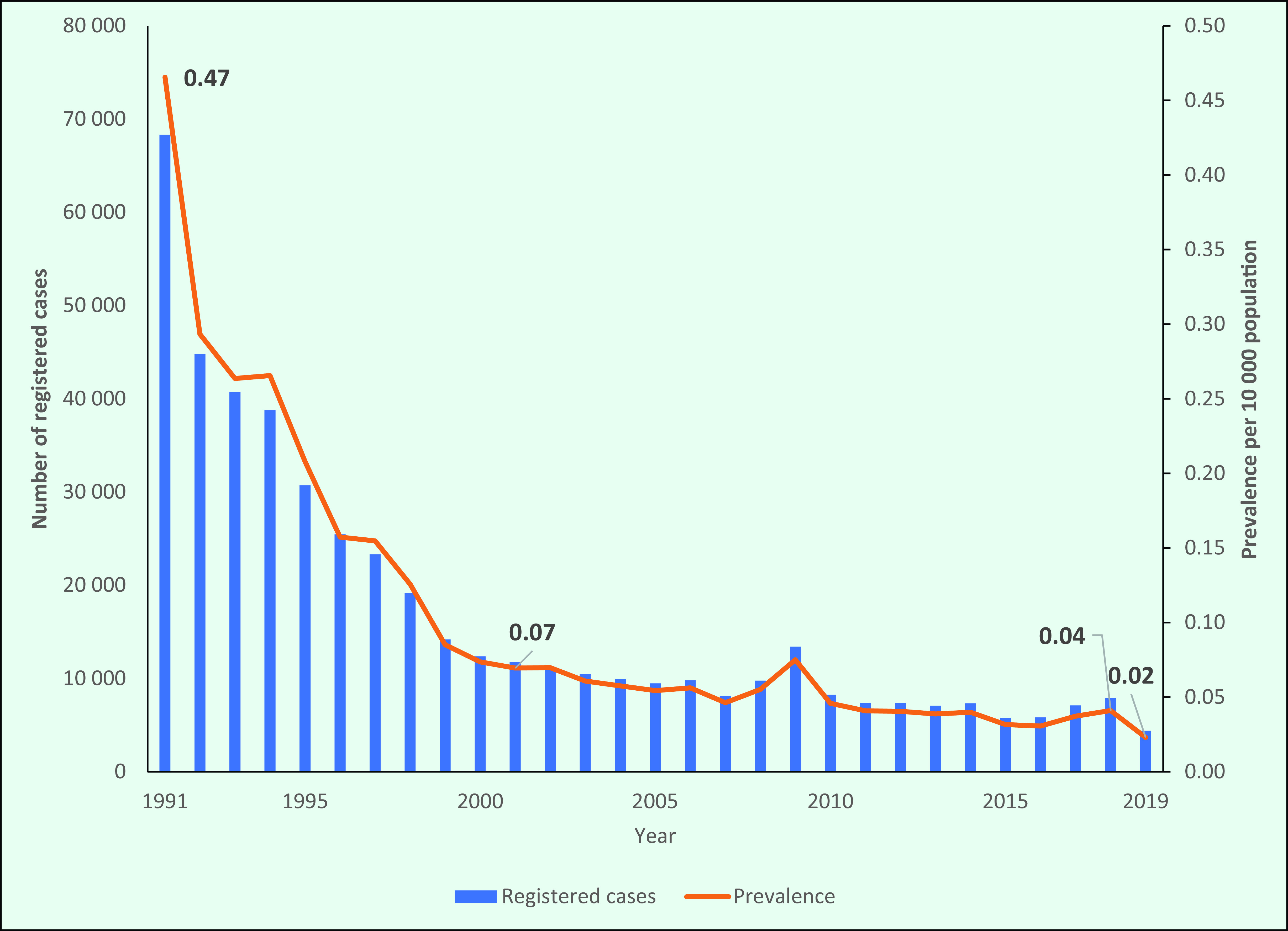
Number of registered cases and prevalence of leprosy in the WHO Western Pacific Region, 1991–2019

The number of registered leprosy cases decreased by 94%, from 68 313 in 1991 to 4381 in 2019 (**Fig. 1**). Most of the decline occurred between 1991 and 2001, when registered cases dropped by almost 83%, from 68 313 in 1991 to 11 757 in 2001. In the following decade, the number of registered leprosy cases continued to decrease, with minor fluctuations. The lowest number of registered cases was in 2019 (4381) (**Fig. 1**).

Similarly, the prevalence of leprosy in the Region declined sharply from 1991 until 2000, from 0.47/10 000 population to 0.02/10 000 population, and it has remained relatively static since 2011, ranging from between 0.04/10 000 and 0.02/10 000 population (**Fig. 1**).

[**insert** Figure 1]

**Figure 2:**
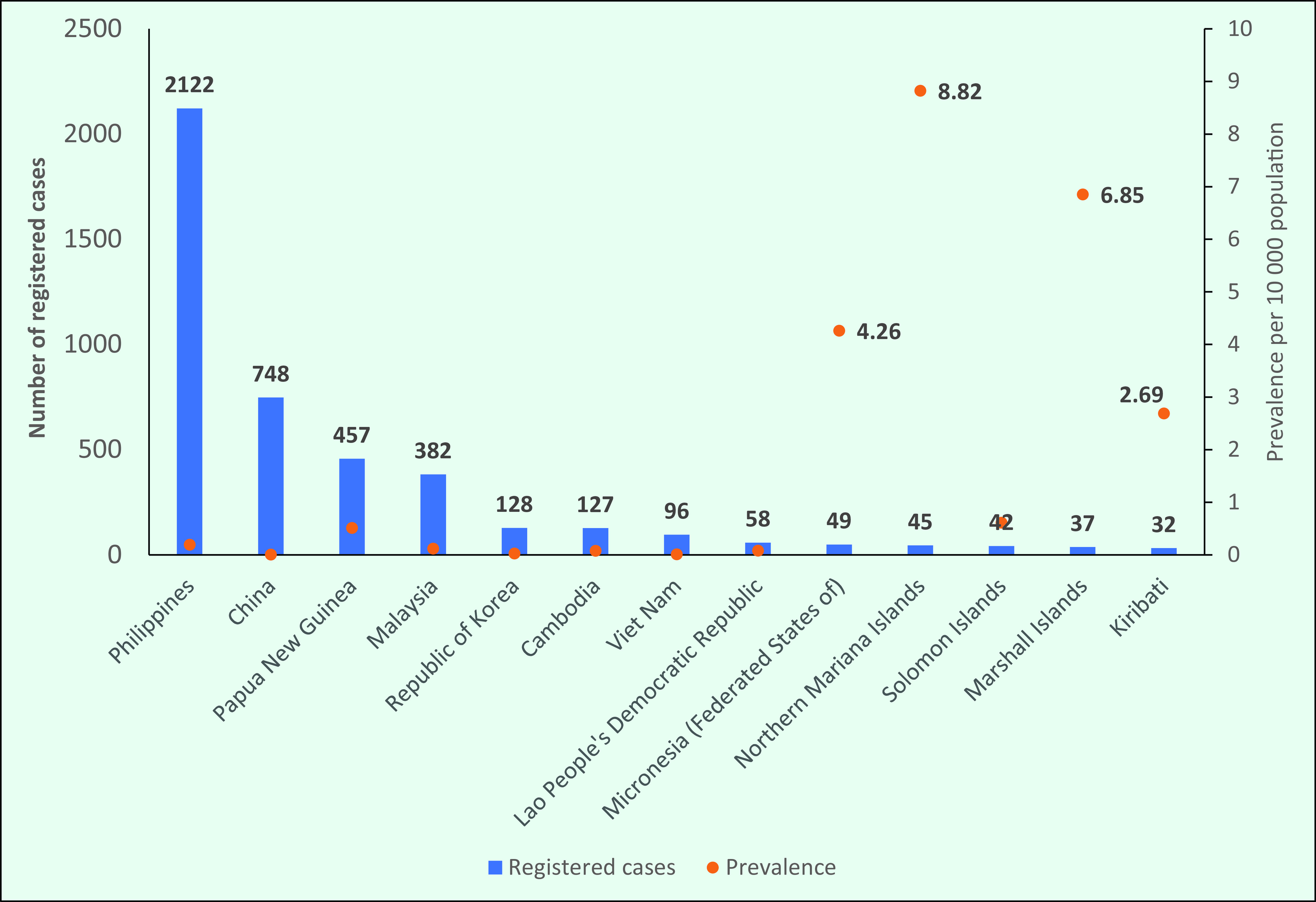
Countries and areas with the highest number of registered cases and prevalence of leprosy in the WHO Western Pacific Region, 2019

In 2019, the highest number of registered cases was reported in the Philippines (*n* = 2122), followed by China (*n* = 748), Papua New Guinea (*n* = 457) and Malaysia (*n* = 382) (**Fig. 2**). Thirteen countries accounted for almost 99% of the prevalent cases in the Region (**Fig. 2**). In 2019, the highest prevalence in a country or area was in the Northern Mariana Islands (8.82/10 000), although this was higher than in previous years: from 2008 to 2018 the rate was consistently < 1/10 000. Other countries and areas with high prevalences in 2019 included the Marshall Islands (6.85/10 000), the Federated States of Micronesia (4.26/10 000) and Kiribati (2.69/10 000); thus, all had prevalences above the elimination level of < 1/10 000.

[**insert** Figure 2]

### New case detection

**Figure 3:**
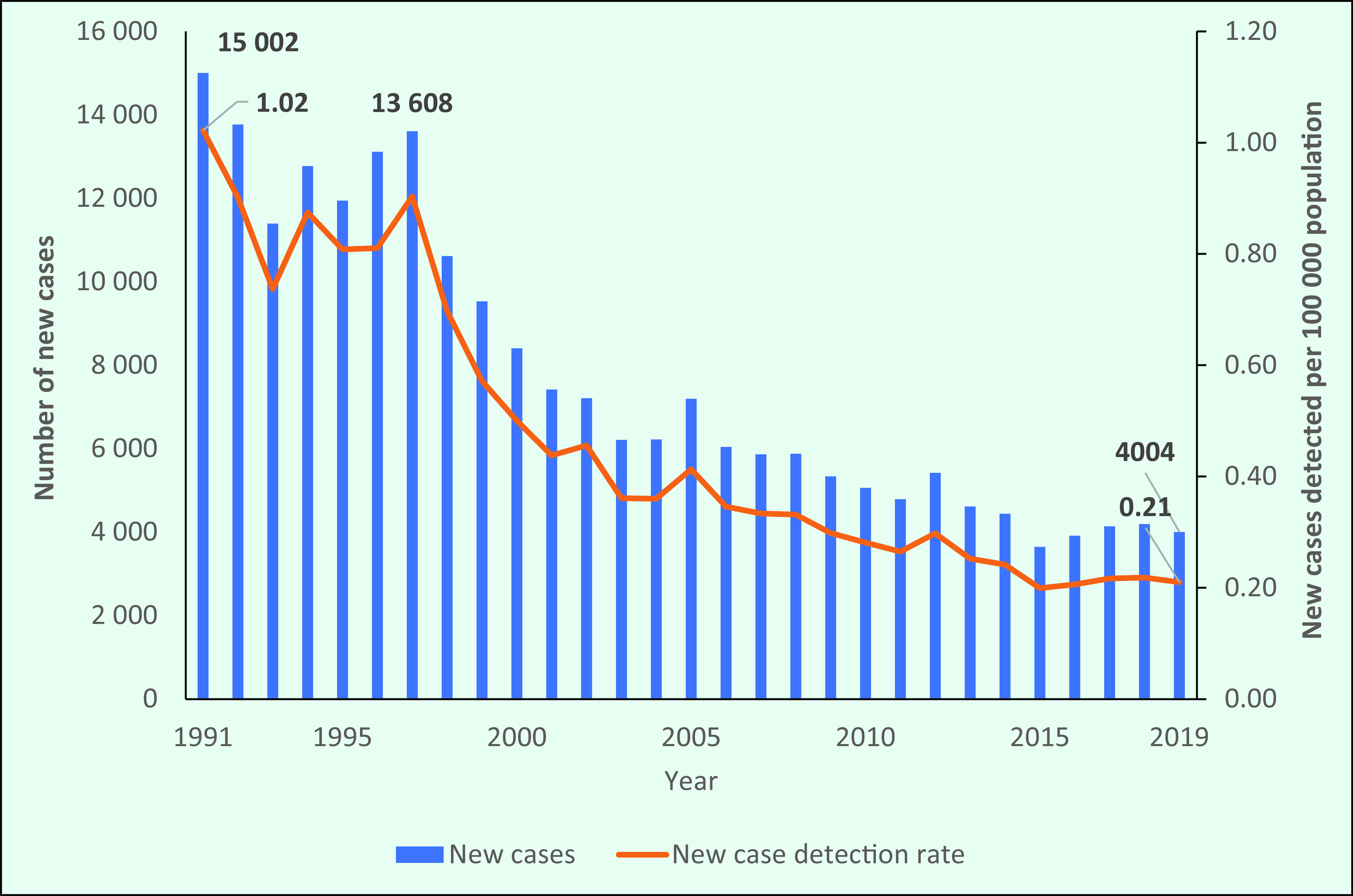
Number of new cases and new case detection rates of leprosy in the WHO Western Pacific Region, 1991–2019

The number of new cases detected in the Region declined by 73%, from 15 002 in 1991 to 4004 in 2019 (**Fig. 3**). In the 6 years from 1991, there were annual fluctuations in the number of new cases and a cumulative decline of 9%. However, from 1997 to 2003, the number of new cases decreased by another 50%, which was followed by a slower decline until 2015; from 2016 to 2018, there was an average annual increase of 5%. Between 2018 and 2019, there was about a 5% reduction in the number of new cases detected (189 fewer cases). Similarly, the NCDR decreased by almost 80%, from 1.02/100 000 population in 1991 to  0.21/100 000 in 2019 (**Fig. 3**).

[**insert** Figure 3]

**Figure 4:**
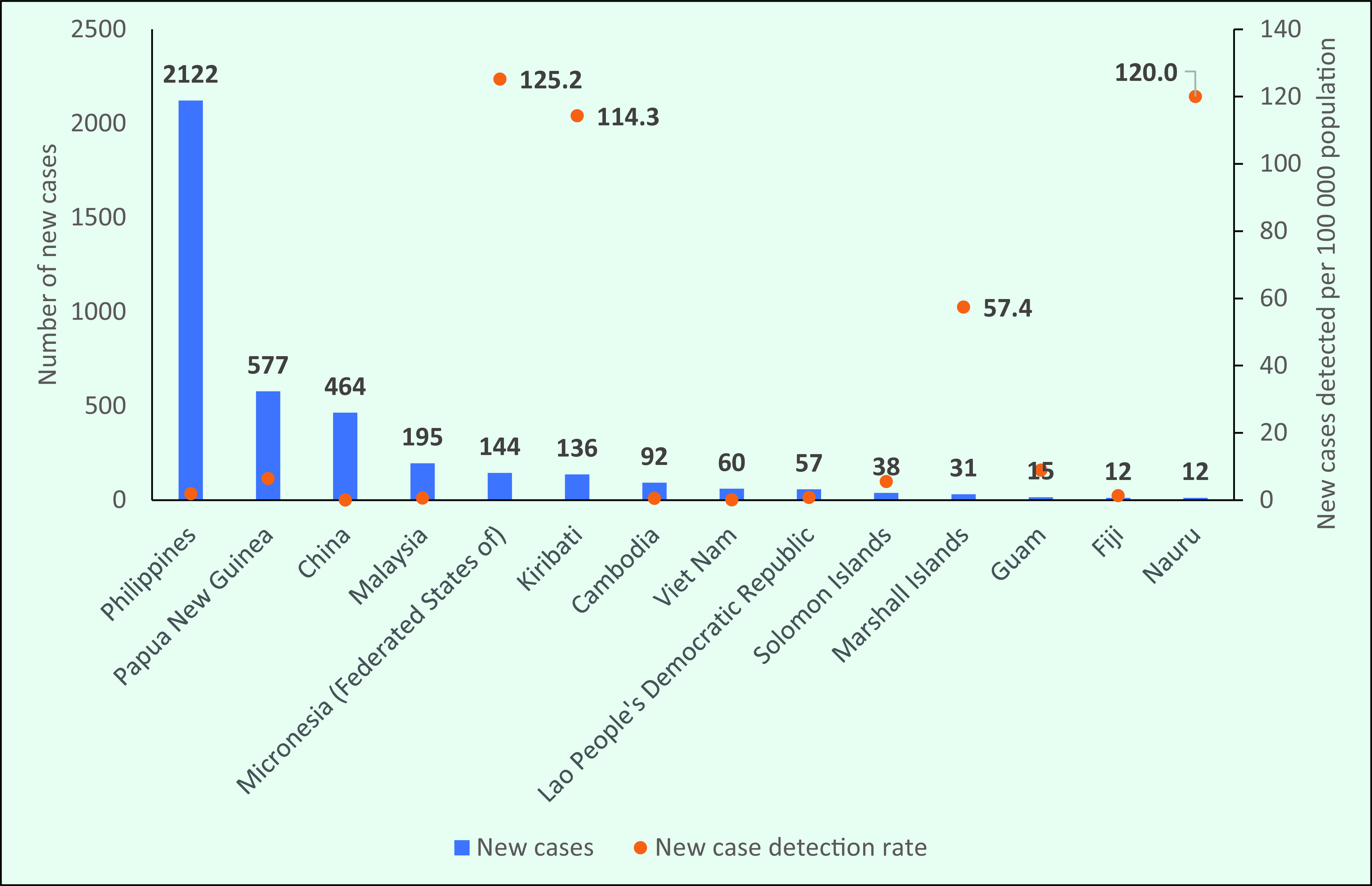
Countries and areas with the highest number of new cases and new case detection rates in the WHO Western Pacific Region, 2019

In 2019, the three countries with the highest number of new cases were the Philippines (*n* = 2122), Papua New Guinea (*n* = 577) and China (*n* = 464) (**Fig. 4**). These countries accounted for 79.0% of the total number of new cases (4004) in the Region.

[**insert** Figure 4]

**Figure 5:**
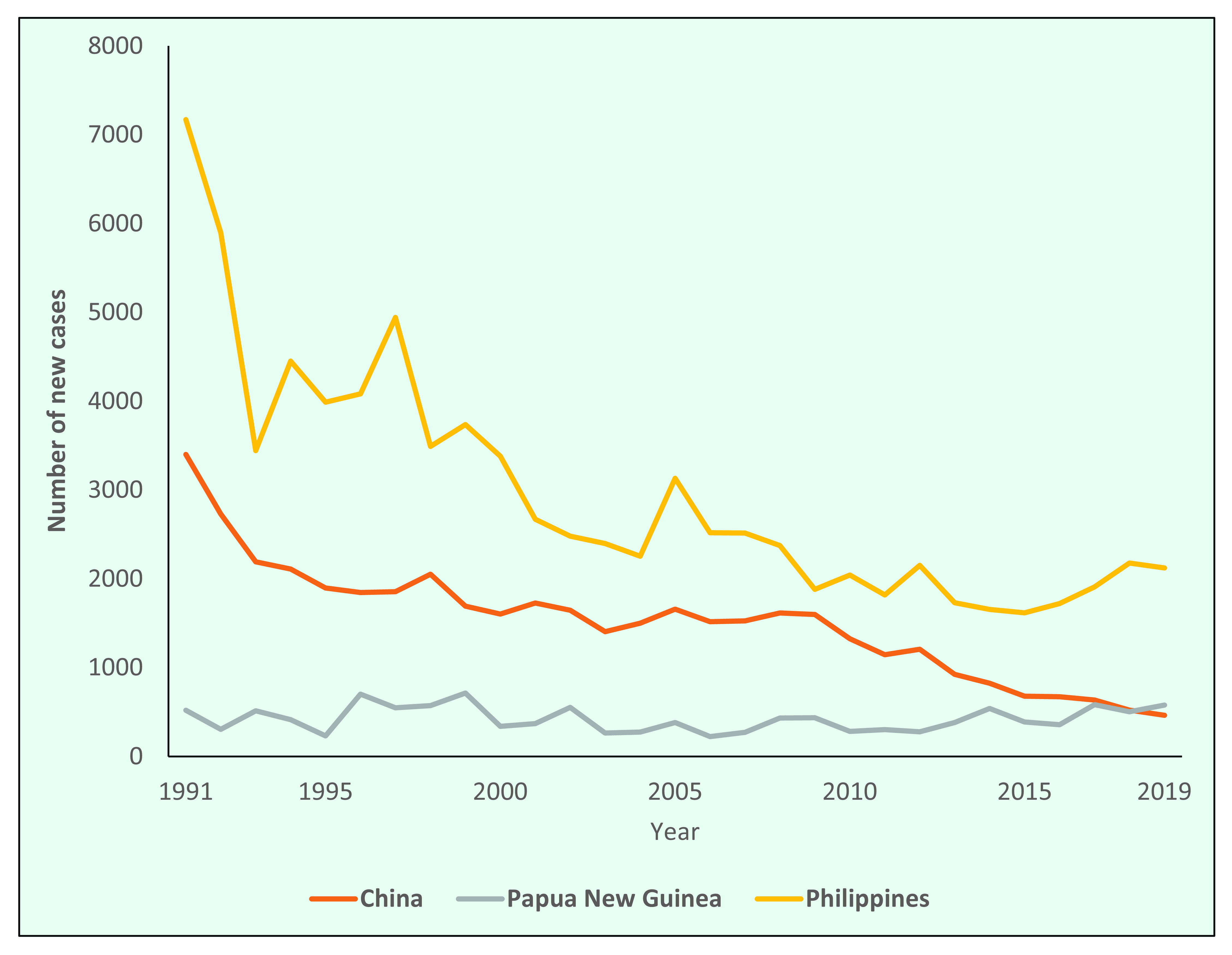
Number of new cases of leprosy in China, Papua New Guinea and the Philippines by year, 1991–2019

Of the three countries with the highest number of new cases in 2019, the number of new cases in China and the Philippines decreased during the study period by 86% (from 3400 in 1991 to 464 in 2019) and 70% (from 7169 in 1991 to 2122 in 2019), respectively, while in Papua New Guinea, the annual number of new cases was 519 in 1991 and 577 in 2019, with fluctuations ranging between 231 and 713. The number of new cases in the Philippines increased during 2017–2019 (**Fig. 5**).

[**insert** Figure 5]

**Figure 6:**
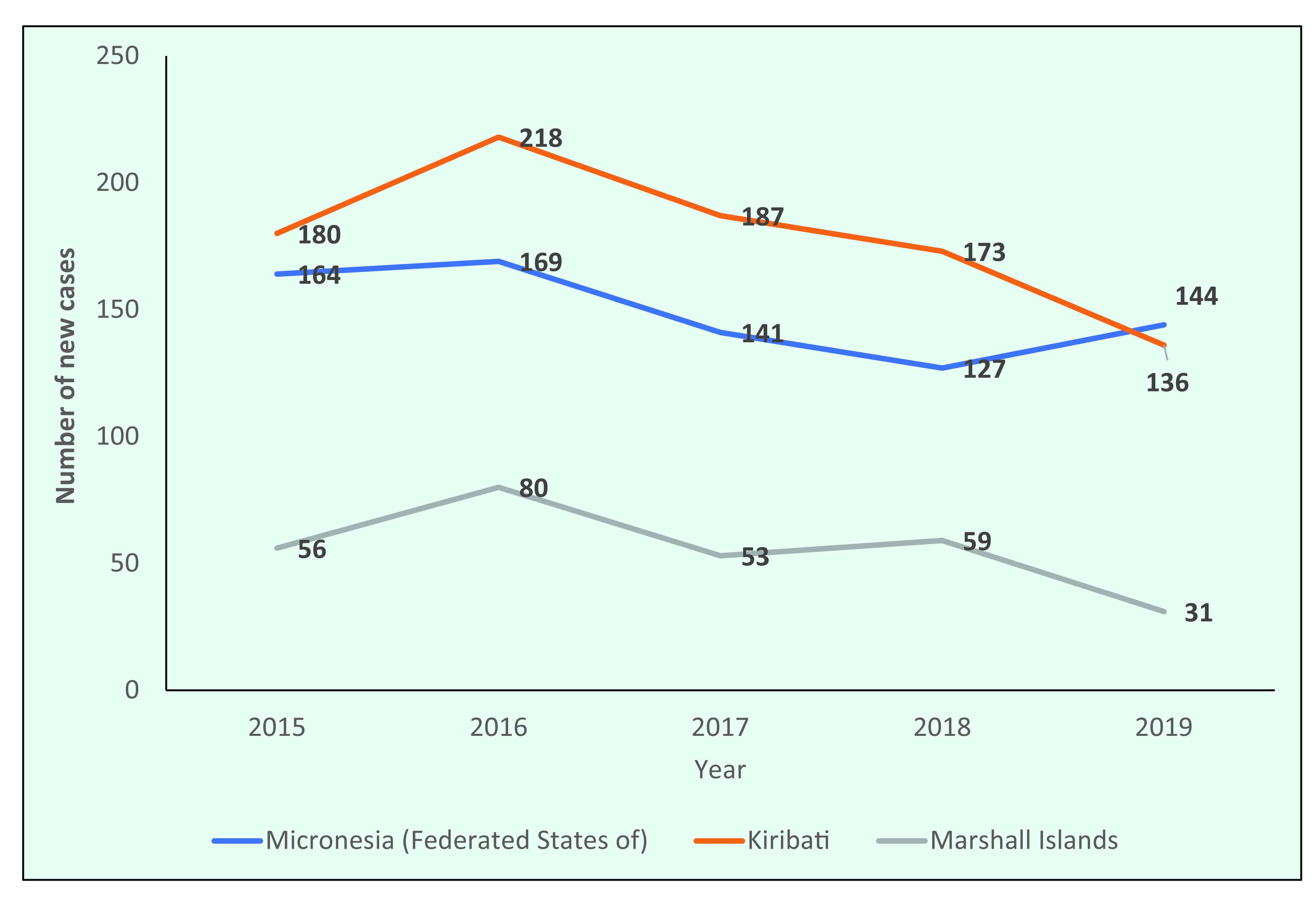
Number of new cases of leprosy in the Federated States of Micronesia, Kiribati and the Marshall Islands, 2015–2019

The number of new cases decreased between 2015 and 2019 in the three countries in the Region that had the highest number of new cases in 2019 (the Federated States of Micronesia, Kiribati and the Marshall Islands) (**Fig. 6**).

[**insert** Figure 6]

### Disease type

The proportion of MB cases in the Region increased from 67.4% (8045/11 943) in 1995 to 85.6% (3428/4004) in 2019 (**Fig. 7a**). In 2007, the proportion of cases (68.3%) reflected a similar decrease to that reported by the Philippines. The overall patterns observed largely reflected those of the three countries with the highest number of new cases (the Philippines, China and Papua New Guinea). Since 2002, the proportion of MB cases in the Philippines has been more than 90% of all new cases detected, while the proportion in China increased from 69% in 1995 to 90.3% in 2019, and in Papua New Guinea the proportion increased from 52.4% to 80.4% during the same period (**Fig. 7a**).

**Figure 7a:**
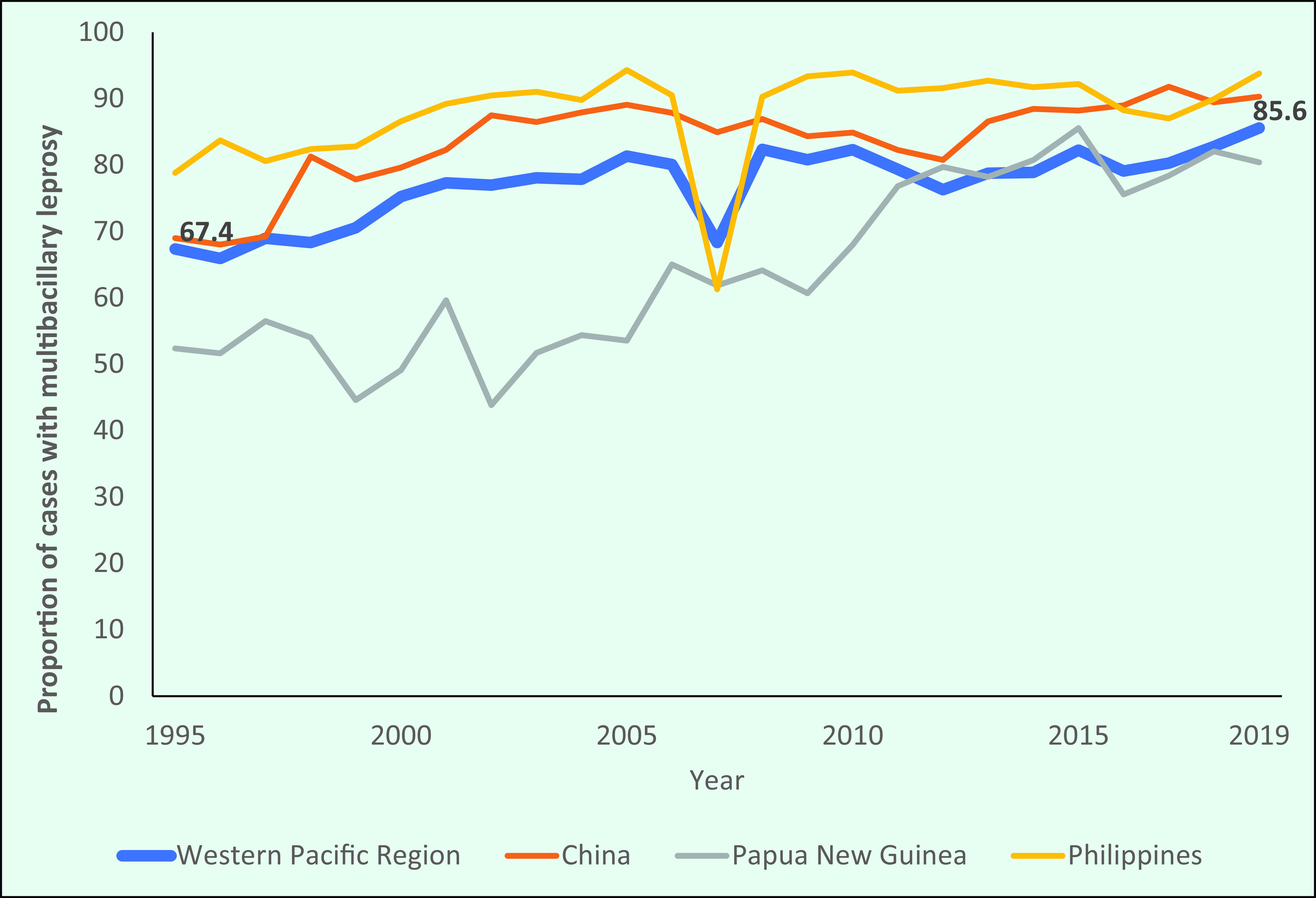
Proportion of new cases of multibacillary leprosy in the WHO Western Pacific Region overall and the three countries with the highest number of new cases in the Region

[**insert** Figure 7a]

In contrast, the three Pacific Island countries with the highest number of new cases reported lower proportions of MB cases. The proportion of MB cases in the Federated States of Micronesia and Kiribati also increased during the study period, from 29.7% (25/84) and 25.4% (15/59), respectively, in 1995 to 47.9% (69/144) and 44.1% (60/136), respectively, in 2019 (**Fig. 7b**).

**Figure 7b:**
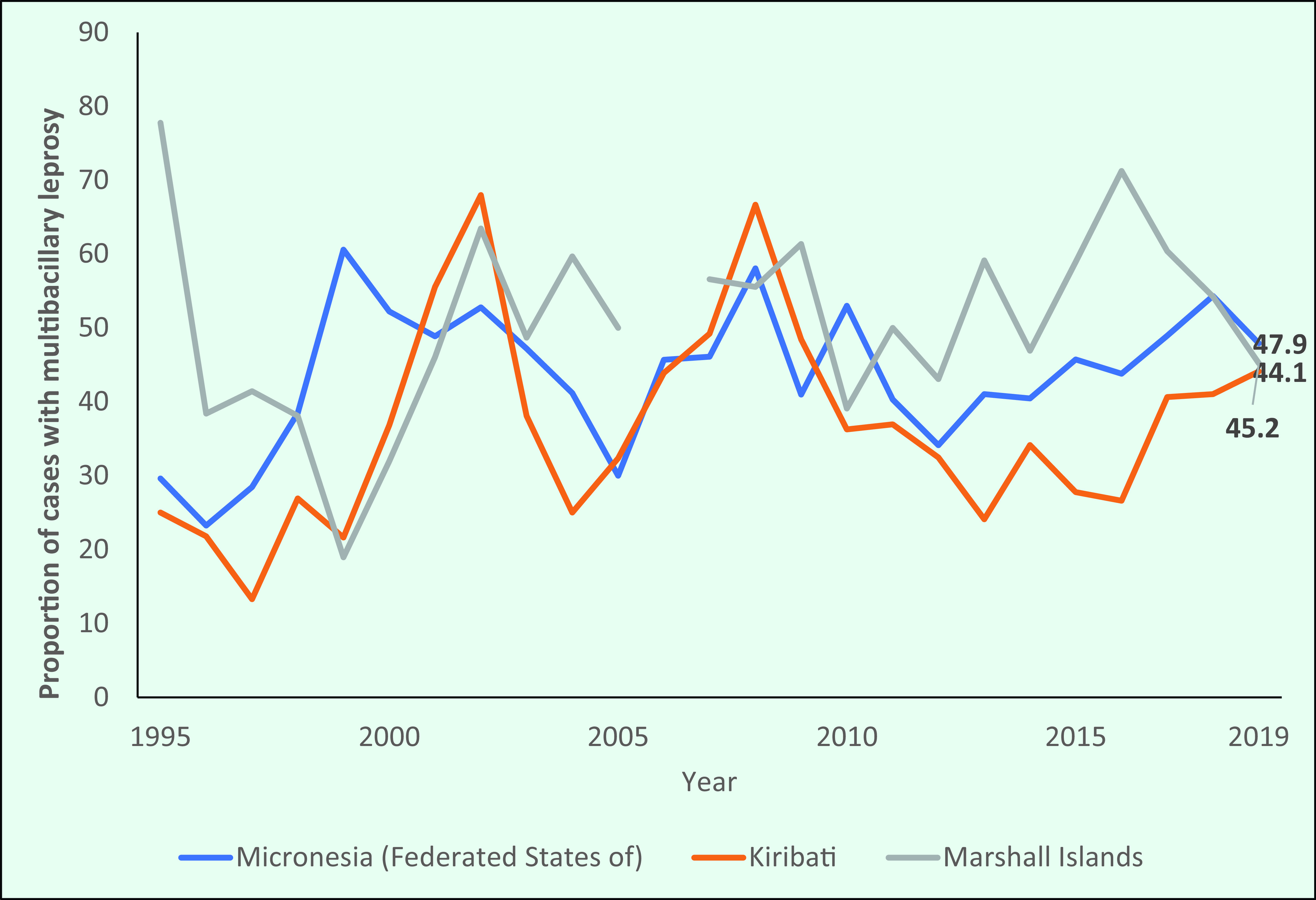
Proportion of new cases of multibacillary leprosy in the Pacific Islands, 1995–2019

[**insert** Figure 7b]

### Leprosy among children

The number of new leprosy cases among children in the Region decreased by 65%, from 1240 cases in 1997 to 424 cases in 2019 (**Fig. 8a**). The proportion of new leprosy cases in children was lowest in 2007 (5.8%; 327/5683) and highest in 2017 (12%; 498/4140).

In the Philippines, the proportion of new cases occurring in children declined from 8.0% in 1997 to 4.7% in 2019, and in China the proportion of new cases in children decreased from 3.4% to 1.3% (**Fig. 8a**). In the Pacific Island countries of the Federated States of Micronesia, Kiribati, the Marshall Islands and Papua New Guinea, the proportion of new cases in children consistently accounted for more than one third of new cases. In 2019, the proportion of new cases occurring among children was 31.9% in the Federated States of Micronesia, 27.2% in Kiribati, 31.2% in Papua New Guinea and 67.7% in the Marshall Islands (**Fig. 8b**).

**Figure 8a:**
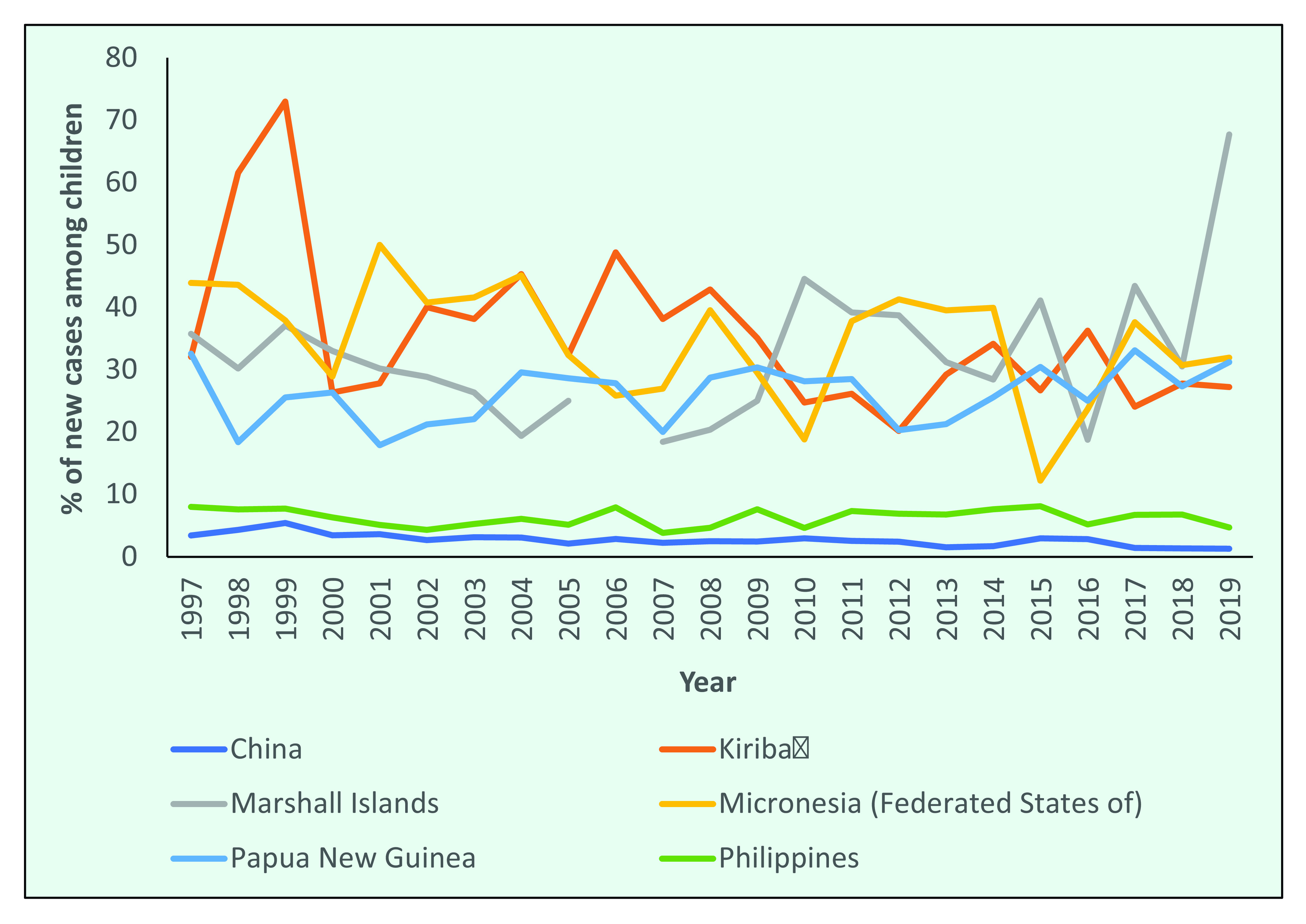
Proportion of new leprosy cases occurring among children in countries in the WHO Western Pacific Region with the highest number of new cases, by year, 1997–2019

[**insert** Figure 8a]

**Figure 8b:**
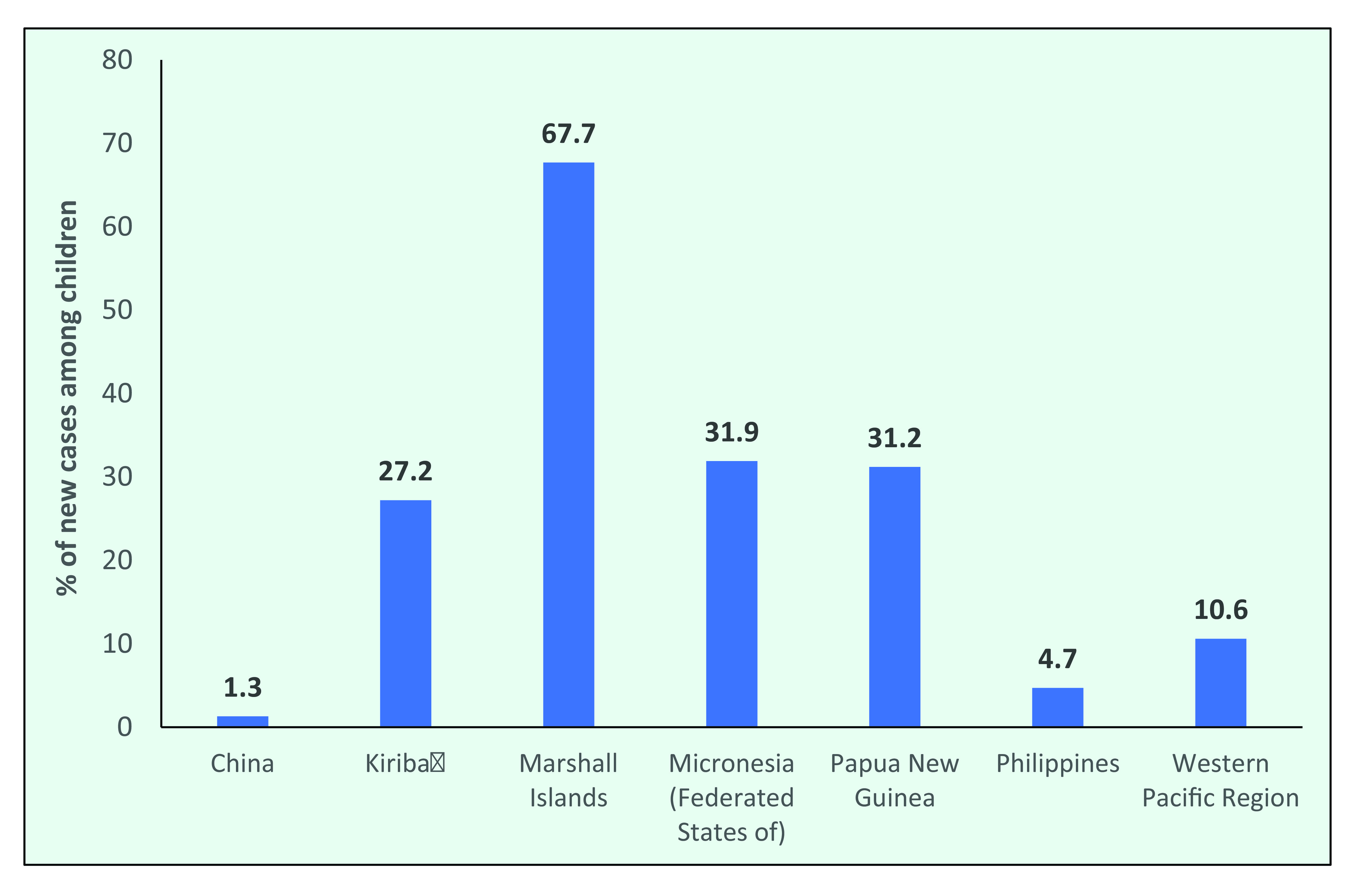
Proportion of new leprosy cases occurring among children in countries in the WHO Western Pacific Region with the highest number of new cases, by year, 2019

[**insert** Figure 8b]

### Leprosy among females

In 2019, the male:female ratio of new leprosy cases in the Region was 2:1. In 2007, the proportion of new cases that were female was 26.8% (1570/5863), which gradually increased to 33.0% (1321/4004) by 2019 (**Fig. 9a**).

Between 2007 and 2019, the proportion of new cases among females in the Federated States of Micronesia and the Philippines increased from 20.6% to 43.8% and from 20.0% to 41.8%, respectively. Kiribati has consistently reported that half of its new cases occur in females, whereas in the Marshall Islands the proportion of females has fluctuated and in 2019 was 54.8% (**Fig. 9b**).

**Figure 9a:**
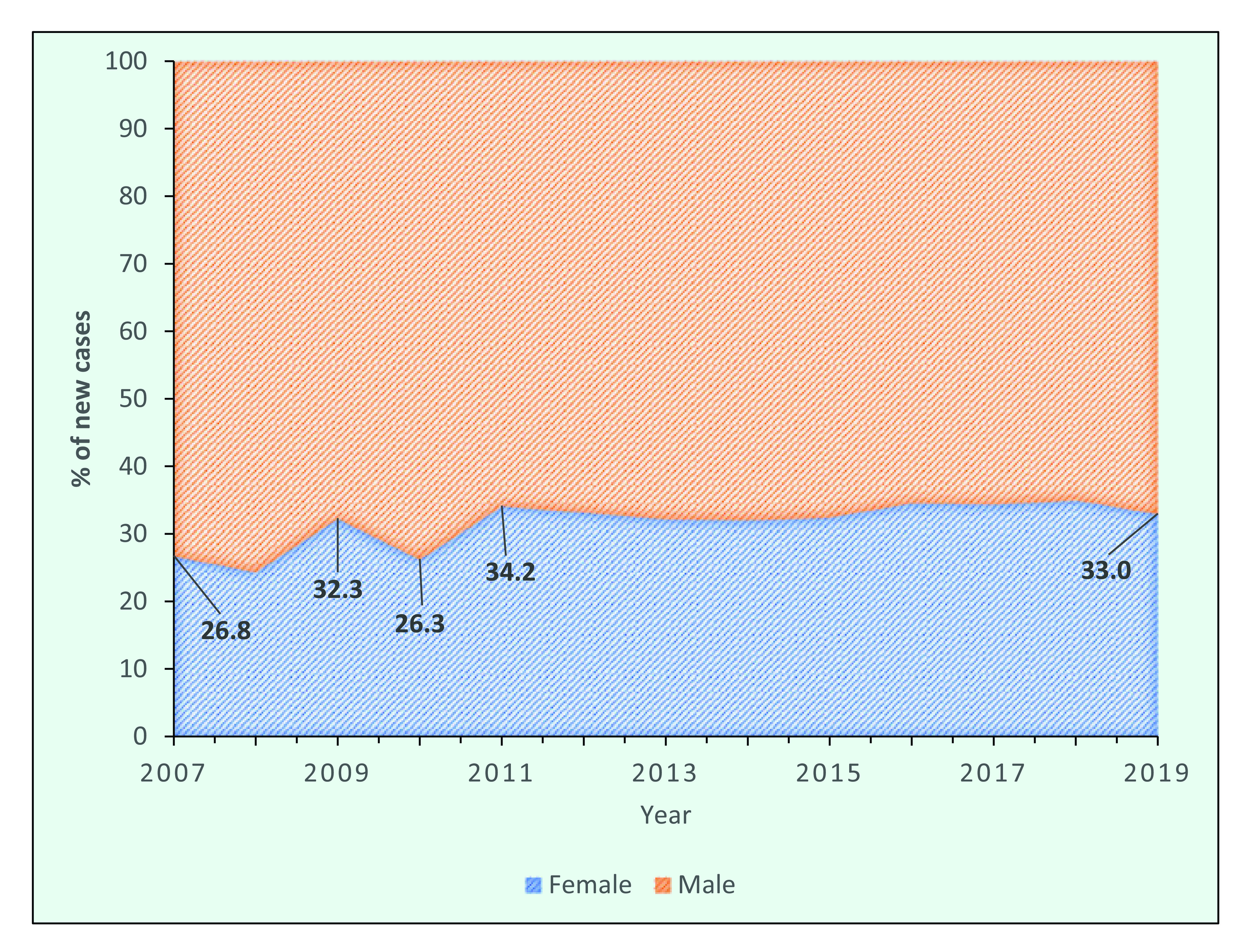
Proportion of new leprosy cases by year and sex in the WHO Western Pacific Region, 2007–2019

[**insert** Figure 9a]

**Figure 9b:**
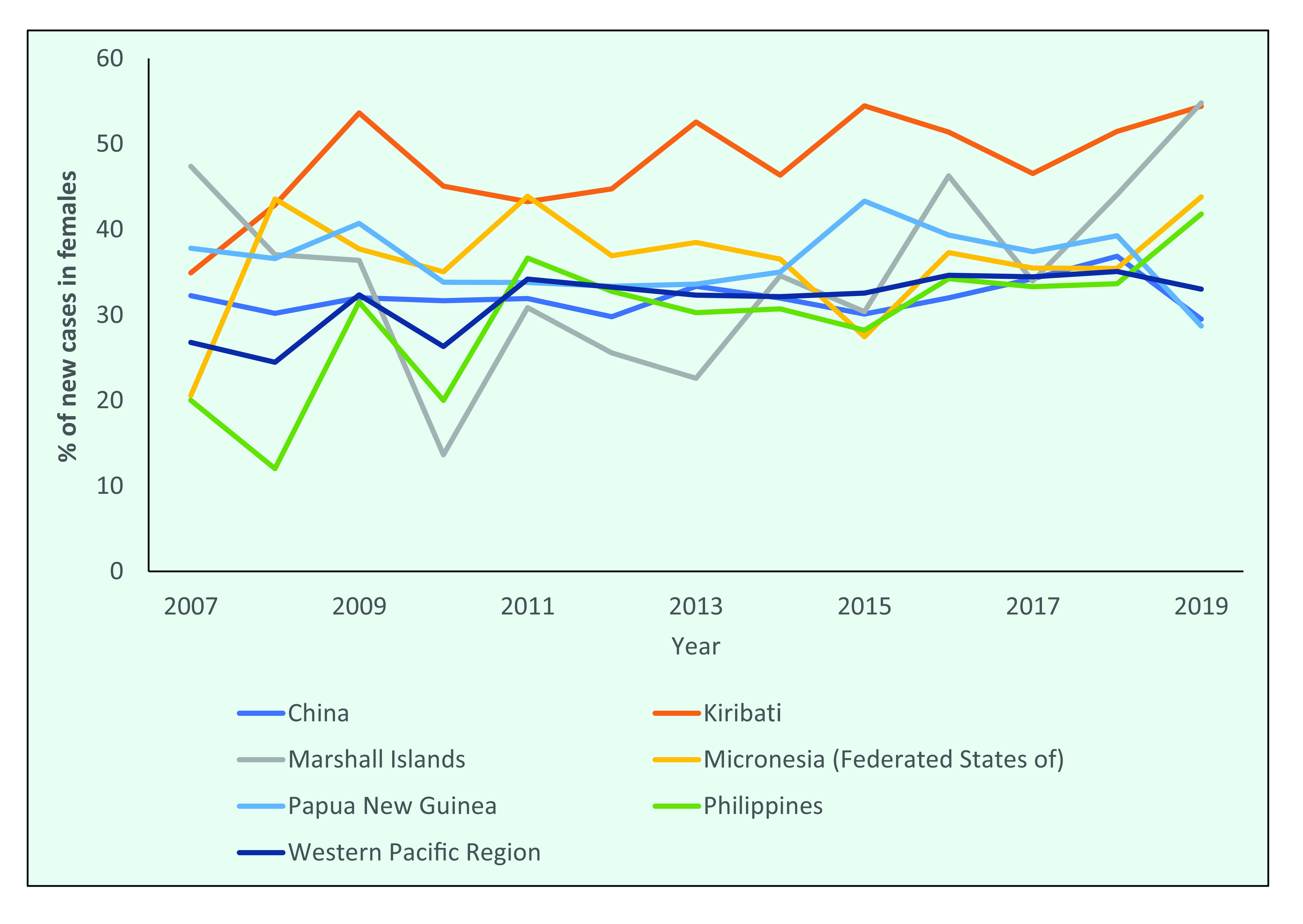
Proportion of new cases occurring in females in the countries with the highest number of new cases, 2007–2019

[**insert** Figure 9b]

### Grade 2 disability

The G2D rate in the Region declined from 1.37/1 million population in 1997 to 0.14/1 million population in 2019 (**Fig. 10a**). The proportion of G2D occurring among new cases also dropped, from 15.2% (890/5863) in 1997 to 6.6% (264/4004) in 2019, with some minor fluctuations in between (**Fig. 10a**). In 1999, the Region achieved the global G2D target of < 1 case/1 million population.

In 2019, the number of new cases with G2D was 264, and the rate was 0.14/1 million. However, in 2019 seven countries and areas in the Region (the Federated States of Micronesia, Fiji, French Polynesia, Guam, Kiribati, Lao People's Democratic Republic and Papua New Guinea) had G2D rates higher than the global target (**Fig. 10b**).

**Figure 10a:**
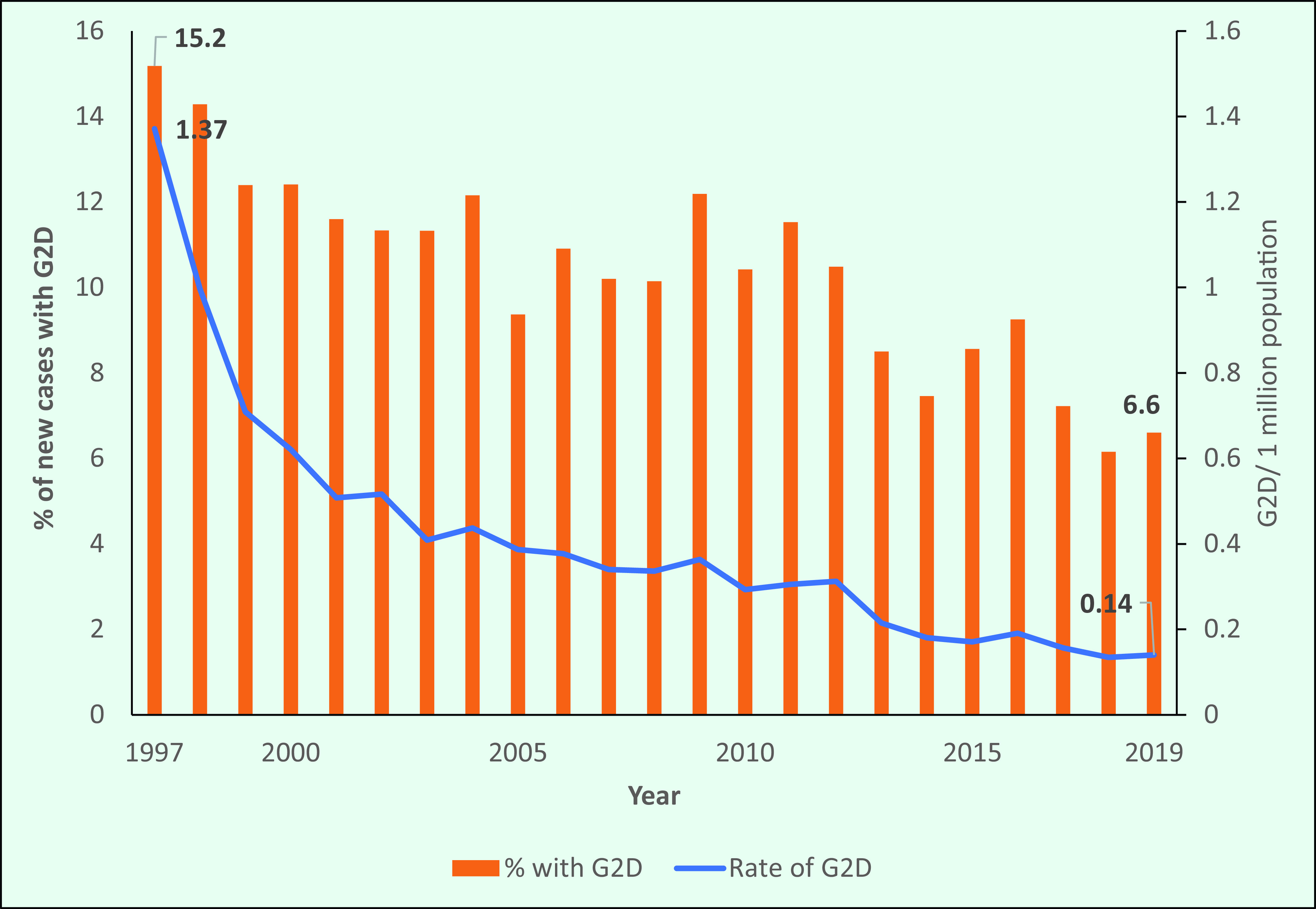
Proportion and rate of new leprosy cases with grade 2 disability (G2D), by year, 1997–2019

[**insert** Figure 10a]

**Figure 10b:**
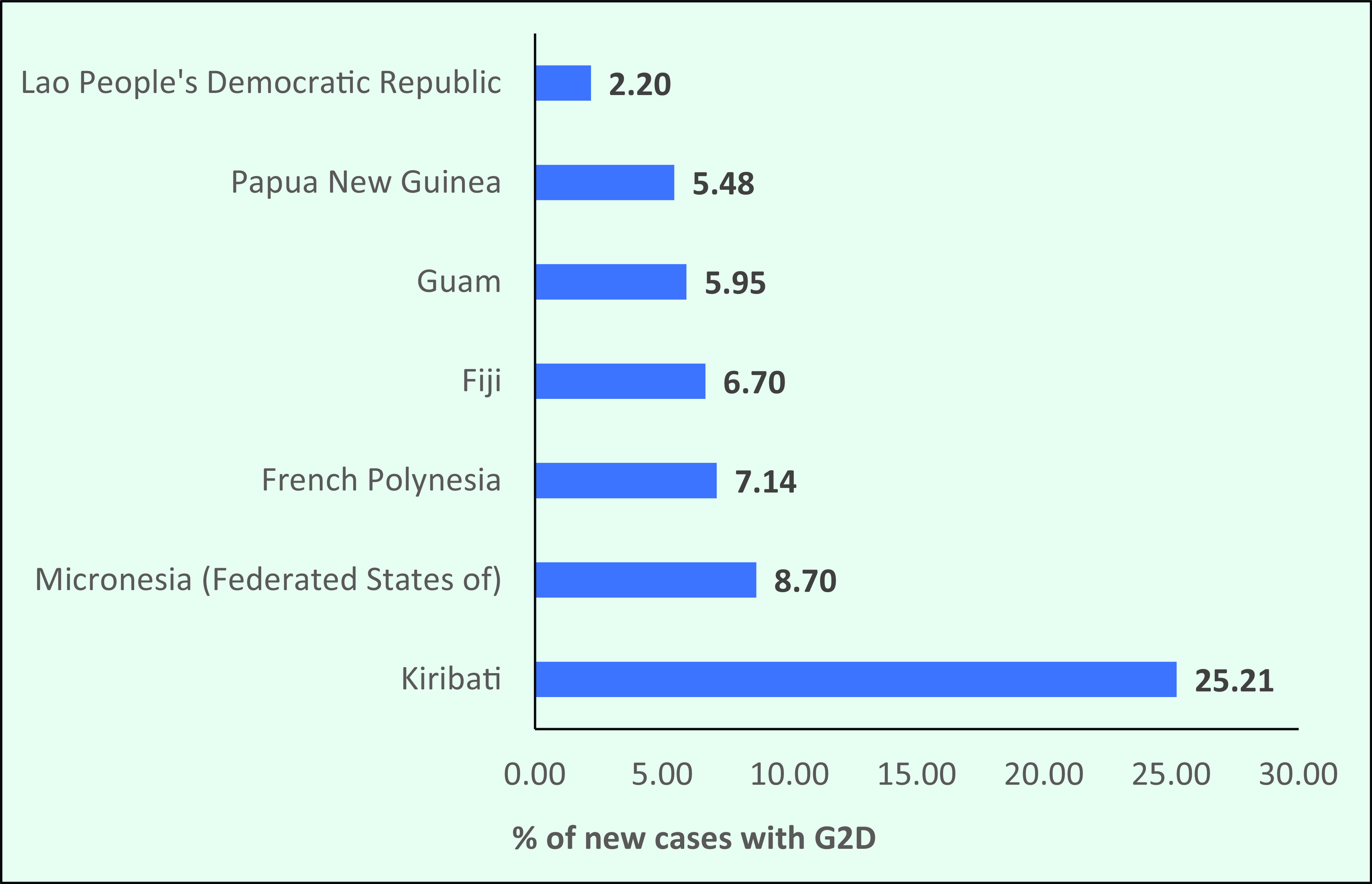
The rate of grade 2 disability among new cases in selected countries, 2019

[**insert** Figure 10b]

There was no reporting of G2D among children before 2016; however, 14 children were reported to have G2D in 2019.

## Discussion

Our analyses provide an overview of the leprosy surveillance data reported to WHO from the Western Pacific Region during nearly three decades. During this time, the prevalence and NCDRs declined regionally, while several countries and areas continued to report high rates and numbers of new cases every year, including cases in children. The proportions of cases of MB leprosy and among females increased during the study period, whereas the rate of G2D and proportion of cases with G2D declined.

The substantial reduction in prevalence and NCDRs of leprosy in the Region demonstrates the success of the regional and national responses to leprosy, particularly during the 1990s. In the second half of the 20th century, leprosy was considered a major public health problem, and in a 1975 survey WHO estimated that there were about 10 million leprosy cases globally and approximately 2 million of those were in the Western Pacific Region. (*9*) In 1981, WHO recommended using MDT for leprosy, comprising a three-drug regimen for MB cases and a two-drug regimen for PB cases. (*2*) The Region adopted MDT by the mid-1980s and completed population coverage by 1994. (*3*) This introduction of effective chemotherapy was likely the main driver of the decrease in leprosy cases in the Region and why the Region achieved the leprosy elimination target by 1988. Given that socioeconomic development and increased health care coverage are also linked with lower leprosy risk, (*10*) these factors may also have contributed to the decrease of cases in the Region.

The 1991 World Health Assembly resolution aimed at eliminating leprosy by the year 2000 (*6*) also may have contributed to global campaigns to reduce the incidence of leprosy, as evidenced by the large decrease in new cases in the years directly after the resolution. This decrease did not occur at the same rate after 2000. This suggests that the final efforts targeting disease elimination are harder to maintain than the initial efforts and that disease elimination requires sustained commitment. The Global Leprosy Strategy 2016–2020 reflects these changes in elimination status, as earlier strategies focused on eliminating leprosy as a public health problem, whereas later strategies emphasized further reducing the burden in areas where elimination had not been achieved. (*6*) Although the leprosy elimination target was achieved regionally in the 1990s, some countries and areas have not reached the target at the national or subnational level. Elimination at the subnational level can be difficult after national-level elimination is achieved. Mobilizing human and financial resources, including national and international partners, to support the implementation of essential activities at the subnational level can be difficult. Finding a way to sustain the political commitment and financing during the post-elimination stage is critical to achieving the vision of a world free of leprosy.

We observed recent fluctuations in the number of new leprosy cases in some countries. This could be explained by ad hoc case-finding efforts in these countries, which largely rely on external funds and other resources, such as mass integrated screening in Ebaye and Majuro in the Marshall Islands and active case-finding in Kiribati undertaken during 2017–2018. A small population can also contribute to fluctuating rates of new cases. The decrease in the overall number of new cases seen during the past 5 years in the Pacific Island countries might be due to programmes continuing routine case-finding and contact tracing and maintaining high treatment completion rates for leprosy cases.

The global decline in the incidence of leprosy may have resulted in a corresponding reduction in awareness of the disease among health workers and in the community. This may translate into delays in diagnosis and treatment and an increase in progression to G2D. Maintaining general awareness about leprosy, sustaining skills among health care workers through basic training and ensuring that there are proper referral mechanisms are essential in areas that continue to report cases. Other possible reasons for the increased proportion of MB cases include ageing populations, as MB disease is more likely to occur in older age groups, undetected MB cases accumulating over the years and being diagnosed during mass screening efforts, shifting from active to passive case detection and using a broader case definition of MB leprosy. (*11*) In high-burden Pacific Island countries, cases may be detected earlier due to ongoing high transmission, which could explain the relatively low proportion of MB disease.

The decrease in the proportion of cases in children, especially in large countries, suggests that transmission is decreasing among the general population; however, case-finding efforts need to be considered. In high-burden Pacific Island countries and Papua New Guinea, the high proportion of cases occurring in children may suggest there is continuing transmission or that active case-finding efforts are targeted at schoolchildren. (*11*) The possibility of an incorrect diagnosis due to the similarity of leprosy with many childhood skin conditions should also be considered, as the majority of cases in children are PB, and eliciting sensory loss in children is challenging.

It is not understood why there is a higher proportion of males with leprosy in most countries, but it could be due to differences in physiology, risk of exposure and access to health care. This last factor may play a part, considering that the proportion of female cases increases remarkably during active case-finding activities. (*7*)

Although the overall proportion of G2D among new cases decreased in the Region during the study period, some countries still have many cases of G2D. There is limited information about rehabilitation services for these cases. The visible impairment that results from leprosy causes stigma and discrimination, as shown by a study conducted in Cebu, the Philippines. The study sought to quantify the impact of the diagnosis of leprosy and of visible impairment on the activities and attitudes of people affected by leprosy. A severe visible impairment was a risk factor for activity limitation and a low level of self-efficacy. (*12*) Careful assessment of all new cases of leprosy for G2D is required because a failure to conduct these assessments may result in a missed diagnosis of G2D. Delayed presentation is a recognized risk factor for disability in leprosy, and it is the result of complex interactions between physical, social, economic and psychological factors. (*13*) Therefore, it is important that countries not only continue active case-finding to diagnose and treat cases early but also assess for G2D in all new cases.

This analysis has several limitations. The number of countries and areas reporting data each year differed, with earlier data not available from many countries and areas. Therefore, the regional-level results require careful interpretation because they were largely influenced by the countries that reported high caseloads. However, this analysis provides useful insights into the regional situation, based on data reported from most of the countries in the Region across nearly three decades.

In conclusion, progress in leprosy control has been possible due to the widespread and free availability of robust MDT, the implementation of good strategies for diagnosis and treatment, strong collaboration with major partners and political commitment from countries where leprosy is endemic. However, leprosy cases occurring among children continue to be reported in the Region, and there are many areas of endemicity at subnational levels. Persons diagnosed with leprosy and their family members continue to face stigma and discrimination, which may act as barriers to early case detection and treatment, limit their opportunities in life and lead to social and economic exclusion. Strong collaboration to ensure universal health coverage using the principles of integrated service delivery and a patient-centred approach will be the keys to ending leprosy. It is essential that strong partnerships are built to address the remaining challenges in diagnosing and treating leprosy. A few countries with relatively higher burdens require more aggressive steps and enhanced commitment and resources to eliminate leprosy.
